# Inhibition of *Lactobacillus fermentum SHY10* on the white membrane production of soaked pickled radish

**DOI:** 10.1002/fsn3.2833

**Published:** 2022-03-21

**Authors:** Yang Yang, Yinyin Lian, Shimei Yin, Huayi Suo, Fankun Zeng, Hongwei Wang, Jiajia Song, Yu Zhang

**Affiliations:** ^1^ 26463 College of Food Science Southwest University Chongqing China; ^2^ 26463 National Teaching Demonstration Center of Food Science and Engineering Southwest University Chongqing China

**Keywords:** bio‐membrane, Inhibition mechanism, *Lactobacillus fermentum*, pickled radish, white film

## Abstract

The formation of white bio‐membrane (shenghua) on the surface of pickle leads to uneatable and spoiled products, which has been the key problem restricting the development of Sichuan pickle industry. In this study, the 17 microorganisms in the white membrane of pickled radish were screened and identified, of which *Candida parapsilosis* was the main strain causing ”shenghua“. The membrane‐forming ability of *Candida parapsilosis* was determined by crystal violet staining to explore its adaptability to the fermentation environment concerning temperature and oxygen. It was found that *Candida parapsilosis* had the strongest membrane‐forming capacity under the aerobic condition at 37°C, with the highest OD_595 nm_ value reached to 3.473 ± 0.07 at 72 h post inoculation. This research identified *Lactobacillus fermentum SHY10* to be the inhibitor of the membrane production of *Candida parapsilosis* via the Oxford cup method on a Petri dish, and via co‐inoculation with *Candida parapsilosis* in pickles. Furthermore, this study specified that the cell‐free supernatant (CFS) of *L. fermentum SHY10* had the most significant inhibitory effects and likely to result from protein substances in the CFS. Proteases treated CFS had significantly reduced inhibitory effects against membrane formation, which confirmed that the active component was protein substances. Overall, this study identified a functional LAB strain with significant inhibitory effects against the white membrane formation in pickles, which provide a safe and consumer‐friendly solution for the membrane problem in the fermented vegetable industry.

## Graphical abstract

This study also explored the adaptability of membrane‐producing strains to the fermentation conditions, namely, temperature and presence of oxygen. Importantly, natural microbes against white membrane formation in pickles were screened and confirmed to be effective in inhibiting the growth of membrane‐forming yeast. The identification and verification of *Lactobacillus fermentum SHY10* being an effective antimembrane bio‐presentative are meaningful to the pickle industry because it is a safe and consumer‐friendly approach to produce high‐quality pickles.

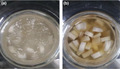

**Practical Application**

This study identified a functional lactic acid bacteria strain with significant protective effects against white membrane formation in pickles. Identifying and verifying the strain are meaningful to the pickle industry that white film has been a costly problem for the fermented vegetable industry. This study provides a safe and consumer‐friendly approach to produce high‐quality pickles.

## 1 INTRODUCTION

Sichuan pickle represents the traditional fermented vegetables in China, and it forms the characteristic Sichuan cuisine that is known worldwide. The spontaneous or semi‐spontaneous fermentation processes of pickles are hard to control. However, pickle production cannot be fully controlled, and nearly 5% of pickle production goes to waste. Nearly 200,000 tons of pickle products are deteriorated in China, resulting in more than 150 million USD yearly economic losses. Among the quality deterioration of Sichuan pickles, the formation of white membranes on the surface of pickles is the most common phenomenon (Feng et al., 2018). White membrane forms under the adverse conditions (temperature, oxygen, pH) of pickle fermentation (Rao et al., 2019).

In commercial production, high salinity pickling was used to control white membrane formation to some extent. However, high salinity pickling has apparent disadvantages as it limits the growth of desirable probiotics and prevents the formation of unique flavors. In this study, strains causing white bio‐membrane in pickles were isolated and identified using Oxford cup and whole‐genome sequencing. Lactic acid bacteria (LABs) have been used as safe bio‐preservatives for various food products, including dairy, meat, aquatic products, fruits, and vegetables (Zhang et al., 2017). Previous research has demonstrated the effectiveness of LABs controlling the growth of undesirable bacteria in fermented products. For example, *Lactobacillus plantarum* AJS2‐4 screened from Jixi sour cowpea, Anhui Province, China was reported to inhibit quorum sensing and bio‐membrane formation of *Aeromonas hydrophila* in sour cowpea (Lin et al., 2019). Anran (2021) found that bacteriocin of *Streptococcus lactis* suppressed the growth of *Listeria monocytogenes* on instant ham and lettuce. Zhao et al. (2019) identified *Pichia guilliermondii* Y35‐1 with significant antibacterial effects on *Colletotrichum gloeosporioides* after harvest. Overall, LABs have good potential as a natural biological preservative in the field of food preservation.

In this study, the bacteria and fungi causing white membrane formation on the surface of pickles were isolated and identified via molecular biological identification techniques. This study also explored the adaptability of membrane‐producing strains to the fermentation conditions, namely, temperature and presence of oxygen. Importantly, natural protection against white membrane formation in pickles was investigated, and *Lactobacillus fermentum SHY10* was screened and identified to be effective in inhibiting the growth of membrane‐forming yeast. The inhibitory efficacy was confirmed both on the plate and in the making of fresh pickles. The identification and verification of *Lactobacillus fermentum SHY10* being an effective antimembrane bio‐presentative are meaningful to the pickle industry. It is a safe and consumer‐friendly approach to produce high‐quality pickles. This study confirmed that effective components are protein substances, while the mechanism of the protective effects of *L. plantarum SHY 10* against “shenghua” remains unclear and requires further exploration.

## 2 MATERIALS AND METHODS

### 2.1 Materials

Fresh raw white radish was washed by DI water, peeled, and chopped into small pieces (approximate 3 × 5 × 5 cm). Chopped radish were transferred to bottles with salty water to achieve 6%–8% salinity. A headspace is left on top of the bottle. The bottles were then sealed and left in a cool and shady place to ferment until the “shenghua” was observed. “Shenghua” pickles were collected from 10 samples of pickled radish from five Chongqing households. The surface of the pickles showed fragmented white bio‐membranes, and the pickles had turbid water and pungent acidic odor. During sampling, the white flowers on the surface of the jar were carefully picked with a glass rod and transferred to a centrifuge tube as sample 1, and 5‐ml pickled vegetable water was taken and transferred to a centrifuge tube as sample 2. No radish was collected. Samples 1 and 2 were brought back to the laboratory in an ice cube box and stored at 4°C for further use. All supplies during sampling are sterilized in advance.

### 2.2 Isolation and identification of membrane‐forming microbes

A small piece of the white membrane was picked by an inoculation ring, mixed in 5‐ml normal saline, then diluted to the appropriate gradient. The 1‐ml pickled water with “shenghua” in sample 2 was taken by a liquid transfer gun, mixed in 9‐ml normal saline, and then diluted to the appropriate gradient. The 100 μl of each culture was spread on MRS medium and YPD medium. Each gradient has triplicates, and all samples were cultured in a 37°C constant temperature incubator (Shanghai Yuejin Medical Device Co., Ltd.) for 48 h. Single colonies were picked and repeatedly streaked and purified until the pure culture was obtained. The colony morphology was observed under a microscope. The single pure colony was selected and inoculated in MRS liquid medium and YPD liquid medium, respectively. Both cultures were incubated at 37°C for 24 h and placed at 4°C for further use.

### 2.3 Bacterial 16S rDNA and yeast 18S rDNA amplification and sequencing identification

The lactic acid bacteria genome was extracted using the Solebao bacterial genomic DNA extraction kit (Beijing solarbio Science and Technology Co., Ltd.). The 16S rDNA fragment was amplified using the bacterial 16S rDNA universal primer 27f as the upstream primer, 1492r as the downstream primer, and genomic DNA as the template. PCR reaction procedures were described below: Pre‐denaturation at 94°C for 3 min for 30 cycles (denaturation at 94°C for 30 s, annealing at 55°C for 30 s, extension at 72°C for 1 min), extension at 72°C for 5 min.

Yeast genome was extracted by Solebo Yeast Genome DNA Extraction Kit (Beijing solarbio Science and Technology Co., Ltd.). Yeast 18S rDNA was amplified with universal primers ITS1 and ITS4, and genomic DNA was used as a template. PCR reaction program was set up as below: Pre‐denatured at 95°C for 5 min, 25 amplification cycles (denaturation at 95°C for 30 s, annealing at 56°C for 30 s, extension at 72°C for 1 min), and elongation at 72°C for 10 min.

The PCR products were then sent to Majorbio Bio‐Pharm Technology Co., Ltd for sequencing. The total reaction volume of the system is 20 μl. Add 2.0 μl 10 × Ex Taq buffer, 1.6 μl 2.5 mM dNTP, 0.5 μl template DNA, One pair of primers each 1 μl, 0.2 μl 5 U Ex Taq, add ddH2O to 20 μl. The obtained sequences were BLAST (https://www.ncbi.nlm.nih.gov/) to perform the nucleic acid data comparison and gene identification.

### 2.4 Isolation and purification of white membrane‐forming strains

The isolated bacteria and yeasts were inoculated into MRS and YPD liquid medium, respectively, and cultured at 37°C. The membrane production on the surface of the microbial liquid was observed and recorded. The microbial isolates with good membrane production were selected, isolated, and cultured at 37°C for 18 h in liquid culture and placed at 4°C until further analyses. The 100‐μl seed solution was inoculated in the 10‐ml sterile liquid culture medium and cultured at 37°C. The absorbance was measured at 600 nm by Ultraviolet–Visible spectrophotometer (Japan Shimadzu Company) every 2 h. The growth curve was drawn with time as abscissa and OD value as ordinate (Cui et al., 2018).

### 2.5 Validation of membrane‐producing ability of test strains

To validate the membrane‐producing ability of test strains, the strain inoculum was added in the pickle‐making process and the pickle samples were observed for membrane (bio‐membrane) formation. The preparation of pickle samples can be summarized as below: cleaning fresh radish → drying → cutting → weighing → vegetable pieces was immersed in 6% (v/v) saltwater (brine) → samples inoculated with the test strain. The strain was cultured to 10^6^ CFU/ml and inoculated into pickles to reach 0.4 % (v/v) concentration of the pickle brine→ fermentation. A control sample following the same procedures was prepared, except the control was not inoculated with the test strain.

### 2.6 Adaptability of membrane‐producing strains to oxygen and temperature

To explore the membrane‐forming conditions concerning oxygen, the selected membrane‐producing strains were inoculated into a liquid culture medium and cultured at both aerobic and anaerobic conditions. The experiment was performed using the dilution spread plate method as described by Du et al. (2016) with slight modification. The membrane formation process was observed, and the colonies were counted at 24, 48, and 72 h, respectively.

Oxygen level and temperature are the two conditions that have not been fully understood and requires further control in the current Chinese pickle processing. To explore the membrane‐forming condition concerning temperature, the selected strains were inoculated into liquid culture medium at 10, 25, and 37°C, respectively, at aerobic conditions using an air penetrable bottle cap. To analyze the effects of oxygen on membrane forming, the selected strains were inoculated into liquid culture medium. One group was air‐tight capped and the other one was not fully sealed and capped with an air penetration cap. The plates were counted and checked for membrane formation at 24, 48, and 72 h, respectively. Crystal violet staining method was used to detect the membrane formation ability. The strain in the logarithmic phase was inoculated into the liquid culture medium, and 200 μl of culture medium was added to each of the 96‐well cell culture plate. The suspension bacteria cells were removed by washing three times with phosphate buffer (0.01 M, PH 7.2 ~ 7.4) at 24, 48, and 72 h post inoculation. The colonies were fixed with methanol for 15 min, then dried at room temperature. The colonies were stained with 0.1% crystal violet solution at room temperature for 30 min. The wells were then gently rinsed with sterile water until the washing solution became colorless, then dried at room temperature. The amount of 100 μl of 33% (v/v) glacial acetic acid was added into each well until it dissolved completely. The OD_595 nm_ values were determined as described previously using the enzyme‐labeled instrument (BioTek Instruments Co., Ltd.) (Liu et al., 2019; Monteiro et al., 2015).

### 2.7 Analysis of the effects of *Lactobacillus fermentum SHY10* supernatant, thallus, and extracellular metabolites on *Candida parapsilosis* B7

*Lactobacillus fermentum SHY10* was inoculated and cultured in MRS liquid medium at 2% (v/v) at 37°C for 48 h. The bacteria culture was then centrifuged at 4°C at 3996 *g* for 15 min, and the supernatant was filtered to obtain cell‐free supernatants (CFS). The CFS was transferred to the culture plates, frozen at ‐80°C, and vacuum freeze‐dried (Beijing Songyuan Huaxing Technology Co., Ltd.) until further analyses. When used, 1 ml of sterile distilled water was added to the culture plate to obtain a concentrated supernatant (Liu et al., 2019). The cells were then collected and divided into two groups. One group was washed twice with sterile water while another group was untreated to obtain the washed and unwashed *Lactobacillus plantarum*, respectively (Liu, 2019).

### 2.8 Effect of proteases on CFS of *Lactobacillus fermentum SHY10*

Four aseptic supernatants of *Lactobacillus plantarum* were prepared and were adjusted to the optimal pH of pepsin, papain, trypsin, and protease K, individually. Each protease was added to an extracted solution adjusted to the protease's corresponding optimal pH to reach the final mass concentration of 1.0 mg/ml. The pH value was then adjusted to the original value of the CFS. The activity of untreated sterile supernatant was determined by Oxford cup method (Peng et al., 2021).

The culture of *Candida parapsilosis* B7 suspension was diluted to approximate 10^6^ CFU/ml using MRS broth. The inoculum of *Candida parapsilosis* B7 was mixed into YPD medium (1% agar) to reach 0.5% (v/v) concentration. The mixture (20 ml) was poured into a YPD plate with a sterilized Oxford cup placed up still in the plate. After cooling, the Oxford cups were taken out carefully to form round holes. Various volumes of *L. plantarum* CFS, unwashed *L. plantarum* suspension, and washed *L. plantarum* suspension were added to the small round holes. MRS medium was used as the control group. Plates were incubated at 37°C for 24 h, and the opaque circle diameters around the well were measured using a ruler. The experiment was performed in triplicate, and the data were recorded at mean ± standard deviation.

### 2.9 Statistical analysis

Excel 2010 software was used to analyze the data. Experiments were performed in triplicate, and results were expressed as the mean value ± standard deviation. GraphPad Prism 7.0 was used to process graphs.

## 3 RESULTS AND DISCUSSION

### 3.1 Isolation and identification of “shenghua” (white membrane) microorganisms

Bacterial sequences were compared with known sequences by the BLAST in the DNA database of NCBI online tool. The results of BLAST identification and homology of strains are shown in Table 1.

**TABLE 1:** Homologous alignment analysis of sequences from isolates

Strain number	BLAST identification results	Homology (%)
1‐2‐4	*Pediococcus ethanolidurans*	100
A2	*Lactobacillus plantarum*	100
A3	*Lactobacillus pentosus*	100
A4	*Paenibacillus barcinonensis*	99.56
A1	*Paenibacillus barcinonensis*	99.49
A6	*Lactobacillus plantarum*	100
A7	*Lactobacillus plantarum*	100
A8	*Paenibacillus barcinonensis*	99.49
A11	*Paenibacillus barcinonensis*	99.49
A12	*Paenibacillus barcinonensis*	99.49
B1	*Bacillus altitudinis*	99.93
B5	*Bacillus altitudinis*	100
B6	*Bacillus altitudinis*	100
B7	*Candida parapsilosis*	99.79
B8	*Enterococcus faecium*	99.65%
B9	*Lactobacillus plantarum*	100.00
B10	*Klebsiella aerogenes*	100.00

A total of 17 isolates were isolated in this experiment, which were identified as *Lactobacillus plantarum* (5 strains), *Pediococcus ethanolidurans* (1 strain), *Paenibacillus barcinonensis* (5 strains), *Bacillus altitudinis* (3 strains), *Enterococcus faecium* (1 strain), *Klebsiella aerogenes* (1 strain), and *Candida parapsilosis* (1 strain). *Lactobacillus plantarum* and *Pediococcus ethanolidurans* are the dominant and characteristic flora of pickles and contributed to the desirable flavors of pickles (Mao et al., 2018; Rao et al., 2018). *Enterococcus faecium* can shorten fermentation time, control nitrite content, and inhibit the growth of spoilage microorganisms (Lu et al., 2017). *Bacillus* are the main spoilage bacteria in pickles. *Bacillus altitudinis* exists in soil, introduced in the final product via unsterilized raw materials (He et al., 2017). *Klebsiella* is related to the bloating bags of pickle products (Ao et al., 2019; Cai et al., 2017). *Candida* widely exists in the environment and can be found on vegetables and soil surfaces that could cause fermented food spoilage (Ao et al., 2019; Zhang et al., 2016).

### 3.2 Screening and validation of membrane‐producing microorganisms

#### 3.2.1 Screening and growth curve of membrane‐producing strains

**FIGURE 1:**
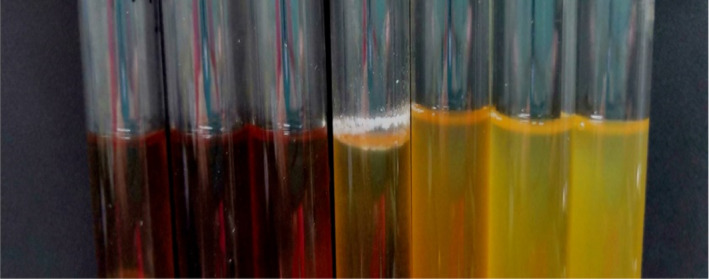
Screening test results of membrane‐producing strains. Note: From left to right: *Pediococcus ethanolidurans*; *Lactobacillus plantarum*; *Enterococcus faecium*; *Candida parapsilosis*; *Bacillus altitudinis*; *Klebsiella aerogenes*; *Paenibacillus barcinonensis*

**FIGURE 2:**
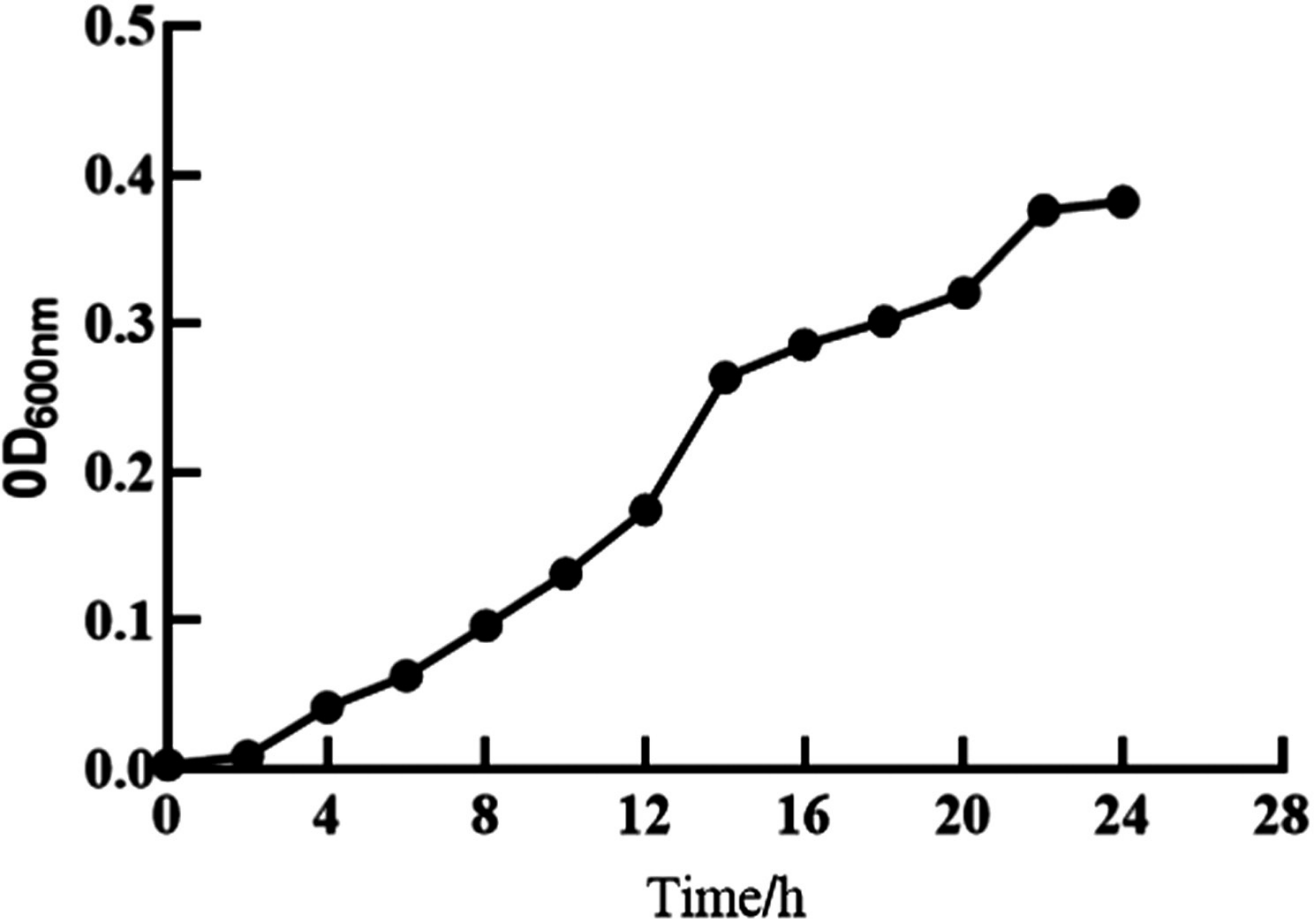
OD values of *Candida parapsilosis during 24‐h incubation at 37*°*C*

The purified strains were inoculated in a liquid medium, and a blank medium was used as a control. The cultures were monitored for bio‐membrane formation, and the strains capable of forming bio‐membrane were selected. As shown in Figure 1, *Candida parapsilosis* began to develop a fragmented bio‐membrane on the liquid surface and the tube wall after 24 h of culture, and a stable thick bio‐membrane was formed on the liquid surface after 48 h of culture. The other six strains did not produce bio‐membrane after 48 h of culture, as shown in Figure 1. The OD values of *Candida parapsilosis* incubated at 37°C are shown in Figure 2.

#### 3.2.2 Verification of membrane‐producing strains

**FIGURE 3:**
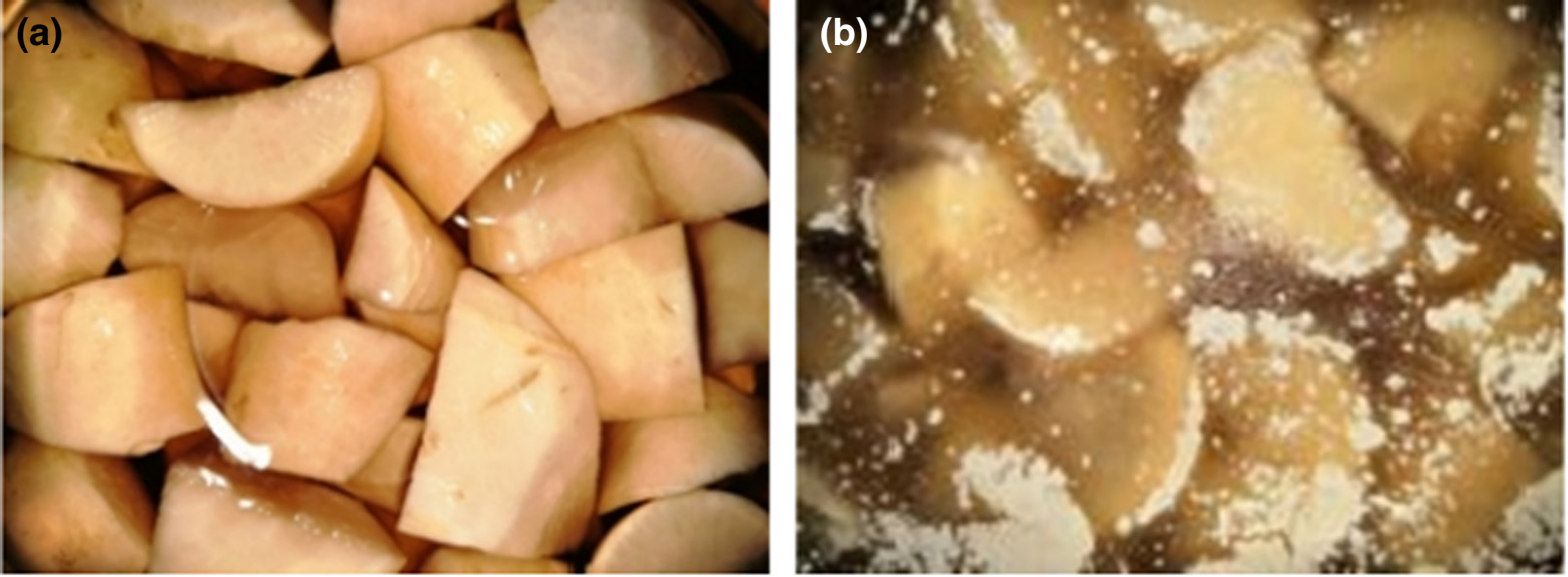
Membrane formation in pickles with/without inoculation of *Candida parapsilosis*

To verify whether *Candida parapsilosis* has the membrane‐producing ability that caused the “shenghua” of pickles, *Candida parapsilosis* was inoculated in fresh pickles, and the pickles without *Candida parapsilosis* inoculation were the control. As seen in Figure 3, the sample inoculated with *Candida parapsilosis* formed visible fragmented white membrane on the surface with a strong sour odor. *Candida parapsilosis* widely exists in the environment and can be found on surface of vegetables. For example, *Candida tropicalis*, *Candida glabrata*, and *Candida valida* lead to the deterioration of fermented vegetables and were previously reported to cause white membrane formation on the surface of fermented vegetables (Lu et al., 2019; Zhang et al., 2016). In this study, *Candida parapsilosis* was found to be the main strain causing pickle “shenghua”.

### 3.3 Adaptability of membrane‐producing strains to fermentation environment

#### 3.3.1 Adaptability of membrane‐producing strains to the presence of oxygen

The colonies of *Candida parapsilosis* were cultured under aerobic and anaerobic conditions, and the numbers of colonies are shown in Table 2. The results showed that *Candida parapsilosis* could grow at both aerobic and anaerobic conditions, but bio‐membrane (white membrane) could only be formed at aerobic conditions (Data not shown). This result corresponded well with Reeser et al. (2007) that aerobic condition promoted the bio‐membrane formation of *Campylobacter jejuni*. Previous studies have identified the transcriptive factors regulating stress response and virulence genes in *Listeria monocytogenes*. For example, SigB is a transcriptive factor that mediates the expression of specific genes and enables them to survive in an oxidative environment. SigB plays an important role in environmental adaptation and bio‐membrane formation for *Listeria monocytogenes* (Zhou et al., 2012). It is reasonable to infer that *Candida parapsilosis* may also have transcriptive factors expressed during the process of environmental adaptation and bio‐membrane formation. It would be necessary to further understand the genetic mechanisms of bio‐membrane formation of *Candida parapsilosis*.

**TABLE 2:** Colony count of *Candida parapsilosis* under different culture conditions

Time (h)	Number of colonies(CFU/ml)	
	Aerobic	Anaerobic
24	4.2 × 10^6^	2.92 × 10^6^
48	3.75 × 10^7^	1.4 × 10^8^
72	8.8 × 10^7^	9.15 × 10^6^

#### 3.3.2 Adaptability of membrane‐producing strains to temperature

The plates spread with *Candida parapsilosis* cultures was incubated at different temperature conditions, and the results of plate counts at different time pointes are given in Table 3. The results showed that *Candida parapsilosis* could grow at different temperatures, while the optimal growing temperature was 37°C. At the same time, the membrane formation of *Candida parapsilosis* at different temperatures was compared (Table 4). The OD_595 nm_ value was the lowest at 10°C, and there was none‐to‐low bio‐membrane formation on the surface of the bacterial supernatant using human eye vision. At 25 and 37°C, there were low visible at 24 h, but visible bio‐membrane formed on the surface of the bacterial liquid at both temperatures at 48 and 72 h. The OD_595 nm_ value was the highest at 37°C, indicating the strongest membrane formation ability at 37°C. The results showed that the bio‐membrane formation on glass surface was highly dependent on culture temperature and time. Interestingly, Bonsaglia et al. (2014) reported that *Listeria monocytogenes* could form bio‐membranes on stainless steel and glass surface at a much lower temperature 4°C, and their report showed that the amount of bio‐membranes increased across the time. This result showed that the cultivation time and temperature play important roles in the formation of bio‐membrane is commonly seen across different bacteria.

**TABLE 3:** Colony counts of *Candida parapsilosis* under different temperature conditions

Time (h)	Number of colonies(CFU/ml)		
	10°C	25°C	37°C
24	1.75 × 10^5^	1.97 × 10^6^	3.20 × 10^6^
48	1.48 × 10^6^	1.44 × 10^7^	3.82 × 10^7^
72	1.86 × 10^6^	7.20 × 10^7^	8.90 × 10^7^

**TABLE 4:** Membrane formation of *Candida parapsilosis* at different temperatures

Time (h)	OD_595nm_		
	10°C	25°C	37°C
24	0.763 ± 0.01	1.988 ± 0.13	2.719 ± 0.14
48	1.588 ± 0.06	3.473 ± 0.07	3.109 ± 0.15
72	2.447 ± 0.07	3.664 ± 0.09	3.582 ± 0.09

The membrane formation of *Candida parapsilosis* was stained by crystal violet, and the membrane formation was quantified and measured as OD_595 nm_. Depending on the absorption value of each microporous plate, the membrane formation was determined to be nonmembrane forming if OD_595 nm_ ≤1, weak membrane forming with 1 <OD_595 nm_ <3, and strong membrane forming with OD_595 nm_ ≥3 (Zhu et al., 2020). The OD_595 nm_ values of *Candida parapsilosis* are given in Table 4. The OD_595 nm_ values firstly increased, then decreased with the highest value 3.473 ± 0.07 at 48 h. At 37°C, The OD_595 nm_ values are greater than 3 at both 48 and 72 h, which indicates a strong membrane formation capacity of *Candida parapsilosis* at optimal temperature.

### 3.4 Antibacterial test of *Lactobacillus fermentum SHY10*

To evaluate the inhibitory effects of *Lactobacillus fermentum SHY10* against membrane formation of *Candida parapsilosis*, *Lactobacillus fermentum SHY10* and *Candida parapsilosis* were inoculated in pickles at the same time as the experimental group, and the pickle samples without *Lactobacillus fermentum SHY10* were used as the control. The fermentation comparison experiments were performed in triplicate.

**FIGURE 4:**
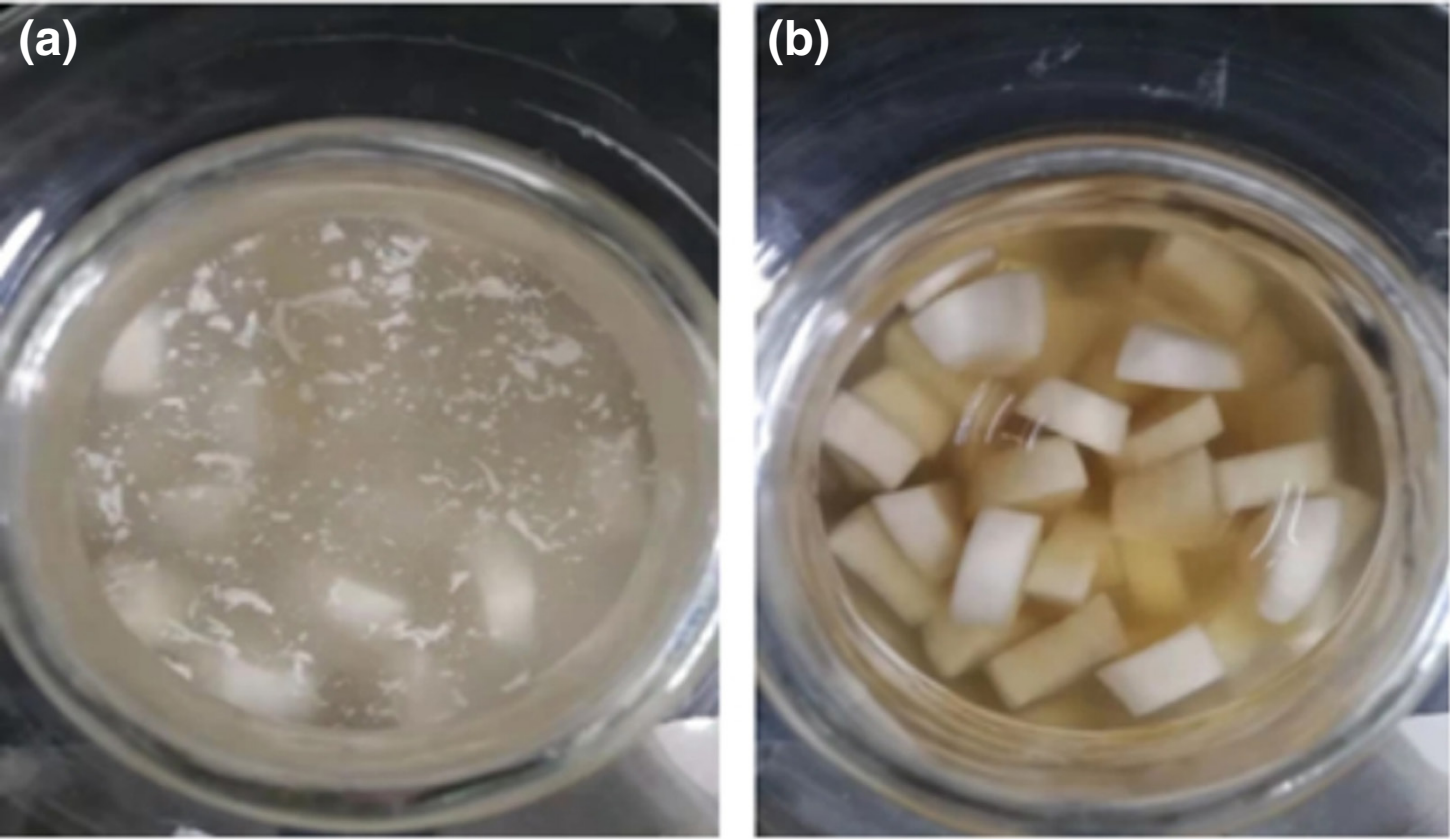
Appearance of pickles inoculated with membrane‐producing yeast (a) and pickles inoculated with both membrane‐producing yeast and *Lactobacillus fermentum SHY10* (b)

A striking visual difference in the amount of white membrane in pickles was observed on day 7 post inoculation as shown in Figures 4a and b. The samples inoculated with *Lactobacillus fermentum SHY10* and *Candida parapsilosis* had a clear transparent supernatant that was comparable to pickles with no *Candida parapsilosis* added (Figure 3a). The results verified the inhibitory effects of *Lactobacillus fermentum SHY10* against the membrane‐forming yeast *Candida parapsilosis* in pickles.

### 3.5 Analysis of inhibitory effects of *Lactobacillus fermentum SHY10* on *Candida parapsilosis* B7

**FIGURE 5:**
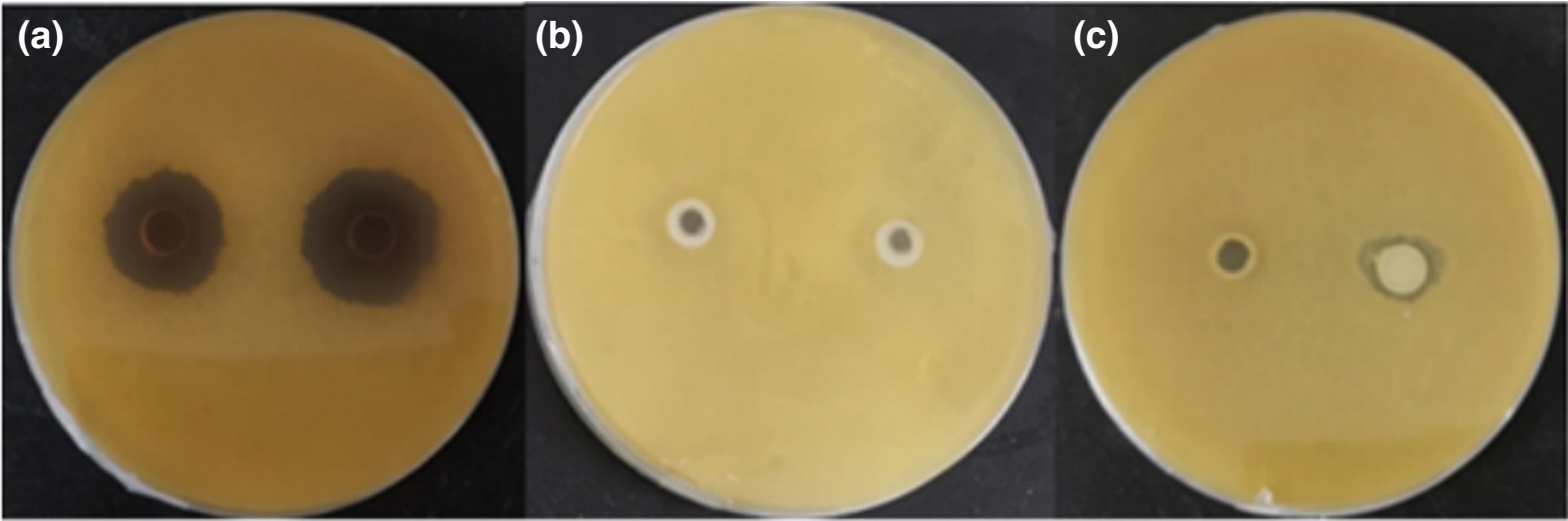
Inhibitory effect of *Lactobacillus fermentum SHY10* on *Candida parapsilosis* on plates

To investigate the antimembrane forming substances of *Lactobacillus fermentum SHY10* CFS, the thallus and extracellular metabolites of thallus of *Lactobacillus fermentum SHY10* were extracted and inoculated with *Candida parapsilosis* B7 using the Oxford cup method as described in 2.8. After 48 h of incubation, the diameters of inhibition zones on YPD plate were measured with a ruler (Table 5). The diameters of inhibition zone increased as the amount of CFS increased, but all volumes showed significant inhibition effects compared to the control (*p *< .05). According to Figure 5a, the CFS of *Lactobacillus fermentum SHY10* had the largest inhibition zone, which indicates the strongest inhibitory effects of extracellular metabolites against the membrane‐forming yeast. The inhibitory effect of thallus with extracellular metabolite on the membrane‐forming yeast was greatly reduced (Figure 5c). The thallus without extracellular metabolites had no visual impact on the growth of the yeast (Figure 5b). Similarly, Song et al. (2018) reported the inhibitory effect of another LAB strain (*L. plantarum* Y42) cell‐free supernatant against the bio‐membrane formation of *Listeria monocytogenes* by interrupting the metabolism of *Listeria monocytogenes*.

**TABLE 5:** Inhibitory effect of various concentrations of sterile supernatant of *Lactobacillus fermentum SHY10* on *Candida parapsilosis* B7

Volume of CFS(μl)	Control (MRS medium)	50	100	150	200	250
Diameter of the transparent circle of suppression circle (cm)	0*	0.47 ± 0.03*	0.82 ± 0.01*	1.14 ± 0.01*	2.32 ± 0.02*	2.61 ± 0.03*

*Indicates significant differences among all date points.

### 3.6 Effect of proteases on CFS of *Lactobacillus fermentum SHY10*

**FIGURE 6:**
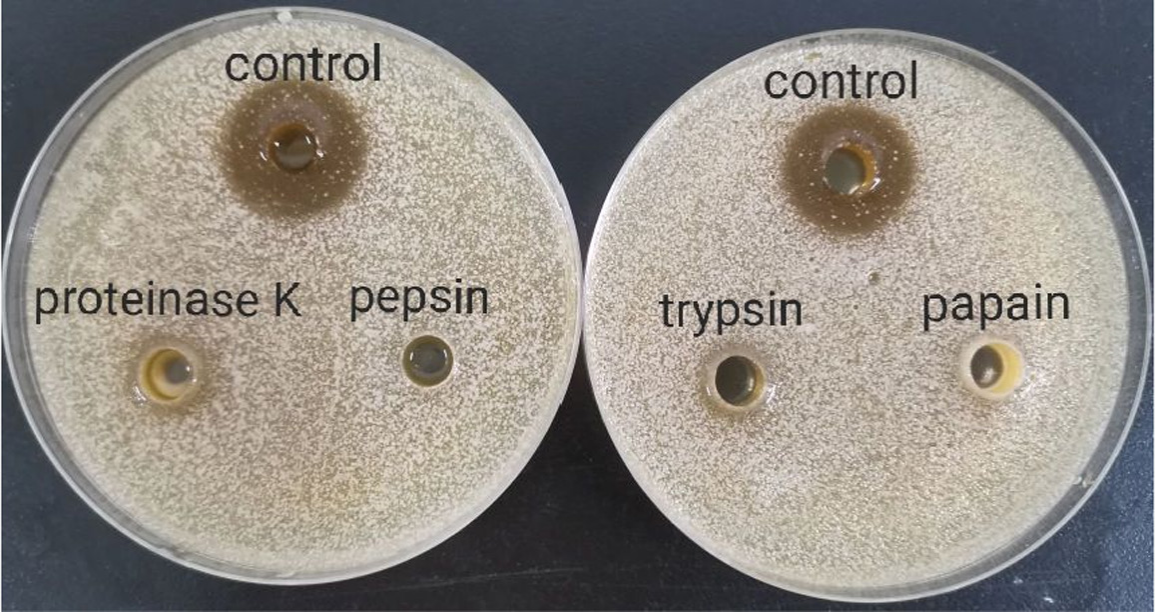
Effect of proteases on the inhibitory effects of supernatant of *Lactobacillus fermentum SHY10* against *Candida parapsilosis*

The effective substances in *L. plantarum* were previously reported to be organic acids and bacteriocin (Wang et al., 2020). Bacteriocins are proteins or antimicrobial peptides that are synthesized by bacterial ribosomes that form channels on the cell membrane of target bacteria or directly inhibit specific functions of target bacterial cells and efficiently exert bactericidal effects (Cleveland et al., 2001). To further investigate if (part of) the antimicrobial components belong to the bacteriocin category and the type(s) of proteins, the CFS of *L. plantarum* was digested by four commonly used proteases against bacteriocin produced by Lactobacillus, namely pepsin, papain, trypsin, and proteinase K (Peng et al., 2021). The digested materials were evaluated for the inhibitory efficacies to explore the type(s) of active components presented in CFS. The supernatant of *L. plantarum* was treated with each protease, separately. Each protease was added at its optimal pH to ensure the maximal efficacy of the enzyme. The proteases treated CFS were used to perform the inhibition zone test against *Candida parapsilosis,* individually. Interestingly, the inhibitory effects were no longer perceived or significantly weakened compared to untreated CFS (control) (Figure 6).

Previous work in our laboratory screened and purified the strain of *Lactobacillus fermentum SHY10* from pickles. *Lactobacillus fermentum SHY10* was found to be effective against *Staphylococcus aureus* due to peptides secreted by the bacteria (Song et al., 2021). Specifically, three antibacterial peptides (ABPs) produced by *Lactobacillus fermentum SHY10* were identified. Among the ABPs, the NQGPLGNAHR peptide showed the most effective antibacterial activity, which may be associated with its interaction with dihydrofolate reductase (DHFR) and DNA gyrase of membrane‐forming microorganisms (Song et al., 2021).

According to Figure 6, the digestion by proteases led to no inhibitory effects. It is reasonable to infer that the functional components against membrane‐forming yeast were protein/peptide substances that have antimicrobial effects. Heredia et al. (2021) found that bacteriocins produced by two *Lactobacillus fermentum* strains isolated from cheese had significant inhibitory effects on various microorganisms, including *Escherichia coli*, *Staphylococcus aureus*, *Listeria innocua*, *Salmonella Typhimurium*, and *Salmonella Choleraesuis*. Previous studies on bacteriocins produced by different lactic acid bacteria were reported. Based on the mechanisms of inhibition, the bacteriocins can be divided into three categories: disruption of the bacterial membrane; inhibition of cell proliferation and division; and destruction of cell wall structure (Kong et al., 2021).

Overall, in this study, the effects of *Lactobacillus fermentum SHY10* against the membrane‐forming yeast were explored and confirmed, and the functional chemical substances were investigated using a protease digestion experiment. The study further confirmed that bacteriocin‐like substances secreted by *Lactobacillus fermentum SHY10* played a key role in preventing the formation of white membrane in pickles. Further researches are required to specify the type of bacteriocin and understand the exact mechanism of inhibition.

## 4 CONCLUSION

In this study, a total of 17 isolates of microorganisms were isolated from “shenghua” pickled radish. The results of molecular biology identification and back inoculation test showed that *Candida parapsilosis* was the main microorganism causing “shenghua” of pickled radish. This study also identified that the optimal conditions for *Candida parapsilosis* to produce bio‐membrane were under aerobic condition at 37°C. *Lactobacillus fermentum SHY10* was explored for the antimembrane formation caused by *Candida parapsilosis* in pickles. This study showed that the effects of inhibition mainly resulted from the CFS supernatant of *Lactobacillus fermentum SHY10*. To further understand the functional substances of membrane inhibition, the CFS was treated with different proteases then tested for inhibitory efficacy. The results showed no inhibitory effect or the inhibitory effect was significantly weakened after the protease treatment, indicating that the active component(s) of its supernatant were likely to be bacteriocin‐like substances.

In summary, the CFS of *Lactobacillus fermentum SHY10* significantly inhibited the growth of *Candida parapsilosis* in pickles. As a microbe naturally exist in fermented products, the protection from *Lactobacillus fermentum SHY10* is a safe treatment to prohibit the growth of white membrane in pickle products. Further studies should focus on the isolation and purification of the functional peptides secreted by *Lactobacillus fermentum SHY10*. Researches should also be performed to understand the mechanism behind the inhibitory effects in order to provide theoretical support for the study of the inhibition of white membrane formation in pickles.

## CONFLICT OF INTEREST

No conflicts of interest need to be claimed by authors.

## Data Availability

The data used to support the findings of this study are available from the corresponding author upon request.
